# Advances in Point-of-Care Testing of microRNAs Based on Portable Instruments and Visual Detection

**DOI:** 10.3390/bios13070747

**Published:** 2023-07-20

**Authors:** Zhong-Yu Wang, Ming-Hui Sun, Qun Zhang, Pei-Feng Li, Kun Wang, Xin-Min Li

**Affiliations:** Institute for Translational Medicine, The Affiliated Hospital of Qingdao University, College of Medicine, Qingdao University, 1 Ningde Road, Qingdao 266073, Chinapeifli@qdu.edu.cn (P.-F.L.); wangk696@qdu.edu.cn (K.W.)

**Keywords:** microRNA (miRNA), point-of-care testing (POCT), visual detection, portable instruments

## Abstract

MicroRNAs (miRNAs) are a class of small noncoding RNAs that are approximately 22 nt in length and regulate gene expression post-transcriptionally. miRNAs play a vital role in both physiological and pathological processes and are regarded as promising biomarkers for cancer, cardiovascular diseases, neurodegenerative diseases, and so on. Accurate detection of miRNA expression level in clinical samples is important for miRNA-guided diagnostics. However, the common miRNA detection approaches like RNA sequencing, qRT-PCR, and miRNA microarray are performed in a professional laboratory with complex intermediate steps and are time-consuming and costly, challenging the miRNA-guided diagnostics. Hence, sensitive, highly specific, rapid, and easy-to-use detection of miRNAs is crucial for clinical diagnosis based on miRNAs. With the advantages of being specific, sensitive, efficient, cost-saving, and easy to operate, point-of-care testing (POCT) has been widely used in the detection of miRNAs. For the first time, we mainly focus on summarizing the research progress in POCT of miRNAs based on portable instruments and visual readout methods. As widely available pocket-size portable instruments and visual detection play important roles in POCT, we provide an all-sided discussion of the principles of these methods and their main limitations and challenges, in order to provide a guide for the development of more accurate, specific, and sensitive POCT methods for miRNA detection.

## 1. Introduction

MicroRNAs (miRNAs) are a type of small noncoding RNA with a length of ~21–25 nt that act as regulators of gene expression at the post-transcriptional level [1]. The miRNA genes are transcribed into hairpin-containing pre-miRNA by RNA polymerase III, and the long dsRNA precursors are processed by Drosha and Dicer consecutively [2,3]. The generated small dsRNAs are loaded onto an argonaute family protein (AGO) to form an RNA-induced silencing complex (RISC). After loading, the passenger strand of the miRNA duplex exits to produce a single-stranded mature miRNA, and the mature RISC induces translational repression, mRNA deadenylation, and mRNA decay [4,5]. miRNAs play vital roles in development. miRNAs regulate cellular activities, including cell growth, differentiation, and apoptosis, and aberrant expression of miRNAs promotes the occurrence and development of diseases. In recent decades, miRNAs have been implicated in various human diseases. Hence, many studies have attempted to apply miRNAs to disease diagnosis, and miRNAs show great promise as diagnostic biomarkers, as miRNAs can not only circulate in the human blood in remarkably stable forms, such as exosomes, but they are also widely present in other bio-microenvironments, such as urine, saliva, and cerebrospinal fluid [6,7]. Accurate detection of dysregulated circulating miRNAs in biofluids is important for miRNA-guided diagnostics in a noninvasive fashion. There have been many conventional methods for the quantitative detection of miRNAs, such as northern blot, microarray, RNA-seq and RT-qPCR [8]. Although these traditional methods are relatively highly sensitive and specific, these approaches also have various limitations. For example, northern blotting and real-time PCR are sensitive and specific, but they are also labor-intensive and require specialized equipment. Microarray and RNA-seq are high-throughput methods that allow the simultaneous detection of multiple miRNAs, but they are also expensive and require complex data analysis, and these approaches for miRNA detection are performed in a professional laboratory, which is challenging for the application of miRNA detection in clinical practice. Therefore, it has driven the development of reliable point-of-care testing (POCT) of miRNAs. Point-of-care testing (POCT) is defined as testing performed near or in the field of a patient, for whom faster results may lead to changes in patient care [9]. Recently, POCT has been applied to the quantitative detection of miRNAs and has made rapid progress. To be more detailed, POCT can provide accurate and ultrasensitive tumor screening results for patients with the advantages of a no-fuss operation, low cost, and rapidity [10,11,12,13]. At the same time, POCT is also suitable for resource-limited areas, or even for self-testing. Previous reviews provided valuable information on the evolution of POCT-detection methods for miRNAs and the applied amplification strategies in POCT for miRNAs [14,15]. The development in detection of multiple miRNAs and the new progress in biosensors, microfluidics, and lateral flow assays (LFAs) for miRNA detection have also been well reviewed [16,17,18]. However, the fast-evolving “miRNA detecting field” needs to be updated, and as far as we know, there is no specific introduction to miRNA detection based on portable instruments and visual detection, while portable instruments and visual detection play important roles in POCT and are very promising methods for POCT for miRNAs with the advantages of portability, convenient readout method, and low price. Therefore, in this review, we summarize recent advances and explore the principles of these methods and give our perspective on future development trends.

## 2. POCT of miRNAs

Point-of-care testing (POCT) is defined as testing conducted near or at the site of the patient, and rapid testing may improve patient care [9]. POCT can provide accurate and ultrasensitive disease screening results for patients with the advantages of easy operation, low cost, rapidity, and a visual readout [10,11,12,13]. The development and validation of POCT for early screening of a series of clinical diseases holds great significance. Moreover, POCT provides the possibility of medical guidance and disease screening in remote areas. Recently, POCT has been applied to the rapid and quantitative detection of miRNAs and has made rapid progress. Microfluidics, paper-based biosensors, portable instruments, and visual detection play important roles in POCT and are very promising methods for POCT of miRNAs. To date, dozens of specialized strategies of miRNA detection based on microfluidics and paper-based biosensors have been reported. Microfluidics and paper-based biosensors for miRNA detection have been well reviewed [17,18]. For details of the research progress of these methods, we refer the reader to the review article recently published. Herein, we mainly focus on summarizing the current progress of POCT for miRNA detection based on portable instruments and visual detection. Based on the comprehensive analysis of such methods, we will explore the possibility and feasibility of developing POCT assays for miRNAs in clinical diagnosis.

### 2.1. POCT of miRNAs Based on Portable Instruments

To avoid the need for bulky instruments and auxiliary devices to obtain a high-sensitivity quantitative signal output, we urgently need a sensing strategy that is controllable, low in cost, and independent of sophisticated equipment but that can offer automated readouts for disease-related miRNAs. In this section, we introduce the current situation of the application of off-the-shelf instruments in miRNA detection, analyze and evaluate the possibility and feasibility of their application, and predict their future development trend. A summary of reported POCT methods for miRNAs based on portable instruments is presented below (Table 1).

#### 2.1.1. miRNA Detection Based on a Personal Glucose Meter

Personal glucose meters (PGMs) are currently among the most widely used POCT devices on the market by virtue of their high portability, low cost, reliable quantitative results, and easy operation [19]. However, in 2011, a PGM was first used to detect non-glucose targets by Lu’s group [20]. This groundbreaking study opens the door to the use of PGMs to measure biomolecules other than glucose.

**Figure 1:**
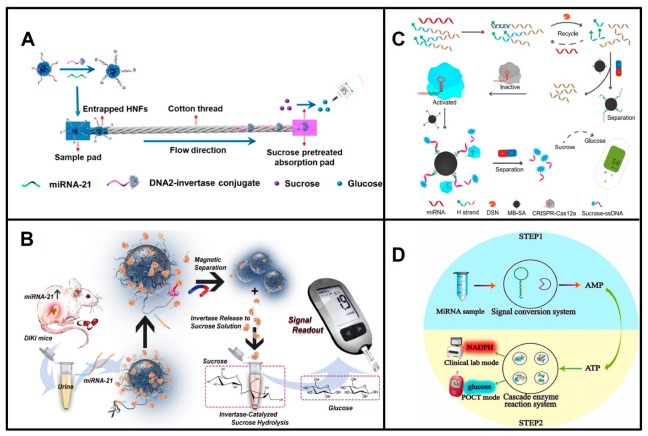
Principles of representative POCT of miRNAs based on PGM. (**A**) Schematic illustration of detection of miRNAs based on HNFs and PGM. Reproduced with permission from reference [21]. Copyright (2018), American Chemical Society. (**B**) Schematic of the releasing of invertase by DNAzyme, and detection of miRNA-21 based on PGM. Reproduced with permission from Reference [25]. Copyright (2020), with permission from Elsevier. (**C**) Schematic of the releasing of sucrase based on DSN-assisted CRISPR-Cas12a, and detection of miRNAs based on PGM. Reproduced with permission from Reference [26]. Copyright (2021), American Chemical Society. (**D**) Schematic of miRNAs produce reporter AMP to trigger the consumption of glucose. Reproduced with permission from Reference [22]. Copyright (2021), with permission from Elsevier.

miRNA was first measured by a glucose meter in 2018 by Wu and his colleagues [21], who designed DNA–Cu_3_(PO_4_)_2_ hybrid nanoflowers (HNFs) that capture the free DNA–invertase conjugates at the start of the cotton thread in the presence of miRNAs and reduce the hydrolysis of sucrose immobilized in the absorption pad into glucose, resulting in a reduction in glucose in proportion to the amount of target miRNAs (Figure 1A). Subsequently, other reports on the detection of miRNAs by PGMs emerged [22,23,24,25,26,27]. They are mainly based on two different principles. One is based on the release or trapping of invertase, amylase, or sucrase, which operates in the presence of miRNAs, resulting in a glucose concentration proportional to the level of the target miRNA. Another strategy is based on miRNAs producing the reporter AMP to trigger the consumption of glucose through enzymatic cascade reactions. In detail, the release of invertase, amylase, or sucrase is triggered by miRNAs through a DNAzyme (Figure 1B), multicomponent nucleic acid enzyme (MNAzyme), DSN, or DSN-assisted CRISPR-Cas12a strategy (Figure 1C) [23,25,26,28]. Pan Fu et al. developed an invertase capture strategy based on dual amplification combining the CHA and HCR reactions, and they achieved measurements as low as 0.36 fM miR-155 [27]. On the other hand, AMP is produced by exonuclease T, which is triggered by miRNAs (Figure 1D) [22]. The exonuclease T-signal transduction strategy [22] and HNFs [21] have relatively low sensitivity because of a lack of signal amplification (Table 1).

Compared with the strategy based on the reporter AMP to trigger the consumption of glucose through enzymatic cascade reactions, the strategy that miRNA operates for the release or trapping of invertase, amylase, or sucrase is more widely used, although the used DNA–enzyme conjugates are costly, and the preparation process is cumbersome. The former holds low sensitivity (Table 1), as it is hard to couple with the nucleic acid amplification strategy. Furthermore, the ATP/AMP in the biofluids will interfere with miRNA levels using this strategy.

The reports indicated that PGMs provide an alternative miRNA detection technology that is affordable, accessible, and easily read using hand-held instruments instead of professional laboratory equipment. However, there is still a long way to go for POCT for miRNAs based on PGMs, such as reducing the operation complexity of DNA-functional MBs or Au-nanoparticles and achieving accurate quantification, before this approach can be applied in clinical diagnosis. The simple and highly efficient “click” chemistry holds great potential for reducing the complexity of DNA-functional MBs or Au-nanoparticle preparation [29]. In addition, the exploration of noncovalent interactions between the DNA/RNA and MBs or Au-nanoparticles could also enable new possibilities in providing potential solutions to address these challenges [30].

#### 2.1.2. Detection of miRNAs Based on a Thermometer

**Figure 2:**
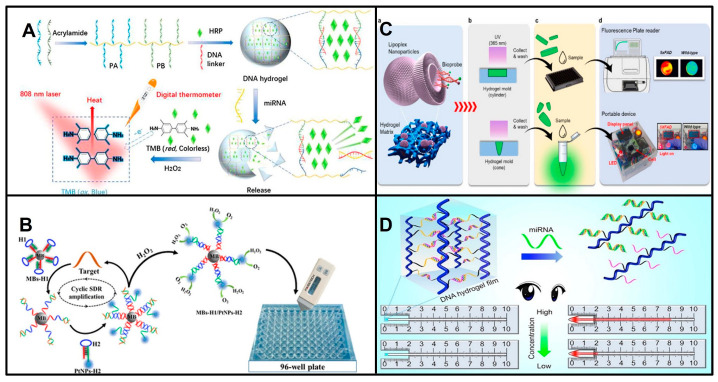
Principles of detecting miRNAs using representative portable instruments. (**A**) Schematic of detection of miRNAs based on (HRP)-encapsulated DNA hydrogel using thermometer. Reproduced with permission from Reference [35]. Copyright (2021) Liu, Zhang, Chen, Cui, Yang, Lu, Qi and Wang. (**B**) Schematic of detection of miRNAs based on pressure meter. Reproduced with permission from Reference [38]. Copyright (2018), American Chemical Society. (**C**) Schematic of detection of miRNAs based on portable fluorometer. (a) The illustration of lipoplex-composite hydrogel platform. (b) Ultraviolet light (365 nm) polymerized CHA probes encapsulated PEGDA based hydrogel matrix. (c) Collecting and washing lipoplex-composite hydrogel. (d) The measurement performed in fluorescence plate reader or portable device. Reproduced with permission from Reference [41]. Copyright (2022), with permission from Elsevier. (**D**) Schematic of detection of miRNAs based on a capillary force meter. Reproduced with permission from Reference [44]. Copyright (2020), with permission from Elsevier.

The thermometer is a widely available, pocket-size quantitative device for temperature measurements. The thermometer has also been demonstrated as a new biosensor system that holds great promise for molecular detection, in which a portable thermometer can measure temperature change representing the presence and concentration of a target molecule. This approach holds the potential to play a major role in environmental monitoring, food safety, and medical diagnostics [31,32]. At present, some reliable studies have applied thermometers to detecting biomolecules, such as proteins [33,34]. Moreover, research has proven that thermometers have great potential in the detection of miRNAs. Liu et al. designed a novel method for miRNA measurement based on a target-responsive horseradish peroxidase (HRP)-encapsulated DNA hydrogel biosensor. The dissociation of the hydrogel is directly controlled by the miRNA, and the released HRP catalyzes the formation of oxTMB from the TMB-H_2_O_2_ system, which exhibits photothermal properties under specific laser irradiation. Thus, the thermometer can realize the visualization and portable quantitative measurement of miRNAs (Figure 2A) [35]. A visualized thermoresponsive sensor based on Fe_3_O_4_ nanoparticles (Fe_3_O_4_ NPs) was developed by the Zhang group and measured miR-141 at the pM level by a thermoresponsive signal [36]. POCT for miRNAs based on thermometers is still at the very early stage. The reported thermometer-based miRNA assays provide convenient readouts. However, there are many challenges that need to be overcome, such as the low sensitivity of the reported thermometer-dependent POCT assays for miRNAs and the complexity of the synthesis of specific nanomaterials, which impede the wide usage of these assays in practice.

#### 2.1.3. Detection of miRNAs Based on a Pressure Meter

A pressure meter, as a portable instrument, is used to monitor the pressure of gases produced by chemical reactions. It is a highly promising candidate for POCT for miRNAs due to its advantages of portability, affordability, and high sensitivity. The biological signal of the target miRNA is converted into a pressure signal generated by the decomposition of the substrate H_2_O_2_ into H_2_O and O_2_, which means that the content of the target can be determined using a convenient pressure meter [37]. Shi et al. developed a POCT method for miRNA detection via a portable pressure meter with a hairpin DNA probe and magnetic separation technology and achieved the first ultrasensitive detection of miRNA-21 (Figure 2B) [38]. Furthermore, for the purpose of the simultaneous detection of multiple miRNAs to improve early cancer diagnosis, Shi et al. combined a portable manometer with a multichannel paper chip. Interestingly, this method uses four DNA tetrahedral probes (DTPs) as capture probes, which improves the signal-to-noise ratio by more than three times compared with that of single-stranded DNA capture probes. Moreover, the ring-oven washing used in this method is cheaper, simpler, and faster than magnetic separation [39]. They achieved the ultrasensitive detection of multiple miRNA targets; however, the complex process of preparing or synthesizing DNA probe-conjugated magnetic beads (MBs) and platinum nanoparticles (PtNPs) presents challenges to their routine clinical usage.

#### 2.1.4. Detection of miRNAs Based on Portable Fluorometers

Fluorescence is the most widely used strategy for the analysis of miRNA biomarkers. In recent years, portable fluorometers have attracted much research interest due to their potential to replace expensive laboratory equipment [40]. Lim and his colleagues reported a portable fluorometer for diagnosing early-stage AD by virtue of miRNAs in the blood as biomarkers (Figure 2C) [41]. They developed a hydrogel-based sensor containing catalytic hairpin assembly (CHA) reaction-based probes. The CHA hairpin probes for miRNA detection were encapsulated in lipid nanoparticles carried by a hydrogel that can be shaped for use in porous plates or 1.5 mL EP tubes and incubated with the test sample to emit a fluorescence signal that can be detected with a portable fluorometer. This fluorometer test was evaluated using the plasma of AD patients and non-AD controls to validate its clinical applicability. Moreover, the main advantage of this advanced method is that it does not require enzymes or temperature changes, which makes it a time-saving POCT diagnostic system with high specificity, accuracy, and sensitivity.

#### 2.1.5. Detection of miRNAs Based on a Capillary Force Meter

A capillary meter is a new type of POCT device based on the principle of capillary action and was first developed by Li [42]. The function of the capillary meter is similar to that of the thermometer, allowing the concentration of the analyte to be read with the naked eye by the height of the liquid column in the vertical capillary tube. Research has already demonstrated the application of capillary meters to detect miRNAs. In 2021, Xueji Zhang and colleagues proposed a visual quantitative meter relying on capillary force alteration in a capillary tube, which converted the wettability variation induced by the target miRNA into a capillary rise height signal [43]. They designed a cyclic amplification experiment in which hydrophobic DNA (single-stranded DNA functionalized with hydrophobic fluorophores) on the inner wall of the capillary was cut to control its wettability, and then the capillary rise height was tested with pure water. The device is used in two steps. First, the capillary is filled with a solution system containing target and accessory DNA molecules to react with the hydrophobic DNA. The target miRNA acts as a trigger, cleaving the hydrophobic DNA on the capillary inner wall and altering its permeability. The solution in the capillary is subsequently washed out, and the capillary is blown dry with nitrogen gas. Second, the capillary is placed vertically in contact with pure water, and the resulting capillary rise height is recorded. The device successfully achieved the measurement of miR-21 in the range of 10^−13^ M to 10^−8^ M in serum with good specificity. Additionally, the device no longer relies excessively on good sealing and the user’s color recognition ability, and the glass capillary used in the device costs less than $0.01, which is much less expensive than other precision chips.

The Li group developed a DNA hydrogel POCT sensor for miRNAs based on capillary force (Figure 2D). The self-driven DNA hydrogel is fixed in film at a capillary tube end. When the sensor is immersed in the solution containing miRNA targets, the probes hybridize with the miRNA targets in the DNA hydrogel sensor, and the permeability of the DNA hydrogel film is increased. Then, the solution flows into the capillary tube, which is pushed by the self-driving action. Thus, the flow-through distance in the capillary tube can quantify the miRNA targets. This POCT assay for miRNAs based on a capillary force meter was reported a few years ago, but its development was hindered by the complexity of capillary tube preparation [44].

#### 2.1.6. Detection of miRNAs Based on Smartphones

Taking advantage of price, wide availability, and pocket size, smartphone-based POCT is emerging as a potential alternative to traditional laboratory-based diagnostic tests [45]. The use of a smartphone’s built-in camera or connected external sensors to obtain information and of various applications to achieve the automatic and rapid analysis of information avoids the traditional use of expensive analysis equipment and the need for professionals [46,47,48]. Therefore, compared with other POCT methods, using smartphones for data or image integration processing can make the whole analysis process more convenient, accurate, visualizable, and adaptive [49]. Smartphones can also share data with internet connections, which is particularly beneficial in resource-limited areas where access to laboratory facilities may be limited or unreliable. Smartphone-based POCTs for miRNAs mostly collect chemiluminescence (CL)/bioluminescence (BL) or fluorescence signals.

**Figure 3:**
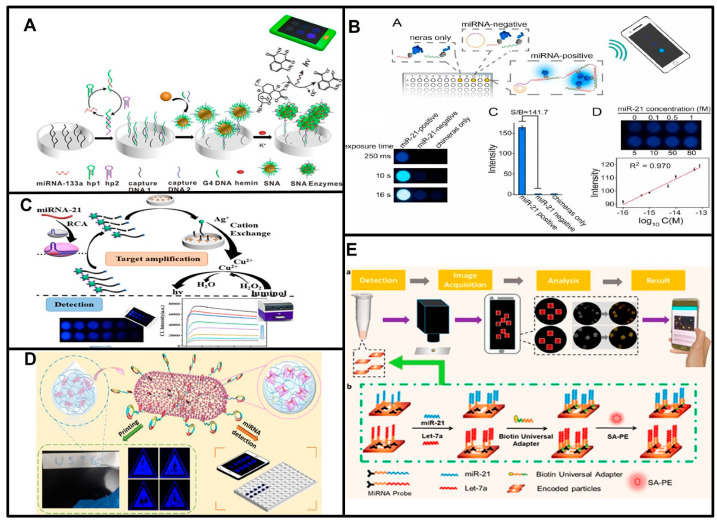
Principles of smartphone-based point-of-care testing for miRNAs. (**A**) Schematic of target miR-133 triggered catalytic harpin assembly (CHA) amplification and captured spherical nucleic acid enzyme. Reproduced with permission from Reference [50]. Copyright (2019), American Chemical Society. (**B**) Schematic of detection of miRNAs based on target triggered-RCA products reassembled the split luciferase-DNA chimeras. Reproduced with permission from Reference [55]. Copyright (2021), with permission from Elsevier. (**C**) Schematic of smartphone detection of miRNA-21 based on CRISPR-Cas12a and cation exchange reaction. Reproduced with permission from Reference [53]. Copyright (2023), American Chemical Society. (**D**) Schematic of G4/MOFzyme system to detect miRNAs. Reproduced with permission from Reference [52]. Copyright (2020), American Chemical Society. (**E**) Schematic of detection of multiple miRNAs based on fluorescence using a smartphone. Reproduced with permission from Reference [49]. Copyright (2019), American Chemical Society.

G-quadruplex/Hemin HRP-mimic enzymes are widely used for chemiluminescence signal production. Sun et al. constructed a novel G-quadruplex/hemin spherical nucleic acid enzyme (SNAzyme)-sensing platform based on chemiluminescence (CL), and the target miR-133 triggered catalytic harpin assembly (CHA) amplification. The CHA products captured spherical nucleic acid enzymes on a 96-well plate, and the chemiluminescence signal was recorded and analyzed by a smartphone (Figure 3A) [50]. Lin Shi and coworkers also reported that the SNAzyme platform detected acute myocardial infarction (AMI)-related miRNAs based on CL using a smartphone [51], G-quadruplex/Hemin HRP-mimic SNAzymes were produced on Au-NPs via target miRNA-triggered DNAzyme cleavage. Although the SNAzyme platform provides high sensitivity, the preparation process is quite time-consuming (Table 2). Lan Mi and coworkers employed the G4/MOFzyme system to detect AMI-related miRNAs, and G4/MOFzyme was assembled when the miRNA-driven RCA products interacted with Zn-hemin MOFs. They achieved CL detection of as little as 1 fM miRNA in human serum by smartphone (Figure 3D) [52].

The CL signal from the Cu^2+^-luminol-H_2_O_2_ system was also used in POCT for miRNAs by smartphone. The Zhang group detected as little as 0.1 fM miR-21. In this strategy, the target miRNA-induced RCA amplification, and the RCA products triggered the trans-cleavage activity of CRISPR-Cas12a to cut linker DNA, which hindered cation exchange between CuS NPs and AgNO_3_, and the chemiluminescence signal was reduced according to the suppression of the Cu^2+^-luminol-H_2_O_2_ system (Figure 3C) [53].

Detecting the BL signal produced from luciferase is also popular in miRNA detection based on smartphones. For example, a luciferase-based logic gate was introduced to sense multiple miRNAs [54]. In principle, the NLuc inhibitor was partially complementary to PNA in the H-Luc-PNA sensor, and in the presence of the target miRNA, the sensor was turned on by a strand-displacement reaction between the target miRNA and PNA in the sensor. The sensor successfully detected three target miRNAs simultaneously. The BL signal can also be turned on with target miRNA-triggered RCA products to reassemble the split luciferase-DNA chimeras. The Wu group demonstrated that this strategy enabled the detection of different miRNAs with attomolar sensitivity and discriminated single-base mutations. It has been successfully used to detect miR-21 and miR-148b in lung cancer patient serum samples using smartphones (Figure 3B) [55]. However, the custom synthesis of the H-Luc-PNA sensor and luciferase-DNA chimeras may hinder its widespread use since the commercial availability and low price of material ensures that the POCT strategy could be widely used.

Instead of recording a straightforward CL/BL signal by smartphone, the Wu group reported a bioluminescent sensing system that integrated bioluminescence resonance energy transfer (BRET) and RCA. The zinc finger protein (ZFP) fused NanoLuc luciferase (donor) and mNeonGreen (acceptor) that were periodically assembled onto the RCA amplicons and produced BRET signals. They detected tumor-associated miRNAs in clinical serum samples [56]. With the advantage of ZFP-fused sensors that are specifically responsive to target-induced RCA products, this flexible platform achieved the detection of multiple miRNA targets without changing the structure of the sensor proteins with high specificity.

The smartphone can also record and analyze fluorescent signals conveniently. Wei Lu et al. designed the fishhook probe-based rolling circle amplification (FP-RCA) assay to integrate the isolation and detection of miRNAs into a compact process, which was simple and effective without the need for bulky and expensive equipment such as a centrifuge, thermal cycler and fluorescence microscope, only a blue light source, and a smartphone camera [57]. Tian et al. developed an intelligent smartphone-based multiple-miRNA detection platform that captured and analyzed fluorescent images (Figure 3E) [49]. In this strategy, the target miRNAs were captured by aptamers immobilized on hydrogel microparticles. After sandwich immunoassays, the target miRNAs were detected through the fluorescent signal of SA-PE on hydrogel microparticles. This method completed the whole process within 2 h. Gao’s group reported a strategy based on photoinduced electron transfer (PET). The target miRNA triggered the production of G-quadruplex/hemin units, the fluorescence of AgNCs was quenched by G-quadruplex/hemin units through PET, and this change was captured by using a smartphone. This method achieved detection of miRNA-21 at a pM level [58]. Sun et al. utilized alkaline-earth sulfide nanodots (ASNDs) as fluorescent labels to successfully measure miR-224 in the range of 10–2000 fM. They employed hybridization chain reaction (HCR) amplification and ASNDs to detect the target miRNA and distinguished single-nucleotide mutations of the target miRNA. The changes in fluorescence can be visualized by the naked eye or captured and analyzed by smartphones [59]. Compared to the self-illuminated CL/BL signal, the excitation of fluorescence requires external light sources, which makes the CL/BL signal more widely applied in POCT for miRNAs.

The emerging POCT assays for miRNAs based on capillary force meters, pressure meters, portable fluorometers, PGMs, and thermometers are still at the very early stage, and most of them reported detection of synthesized or extracted miRNAs. While strategies based on smartphones were more widely reported in the detection of clinical serum samples, which means they have strong anti-interference ability, POCT assays for miRNAs based on smartphones showed higher sensitivity than the others. However, all POCT methods based on portable instruments could provide innovative methods for revolutionizing the field of disease diagnosis only if the problems of complexity and time-consumption in the preparation of DNA/RNA functional nanoparticles are solved and the sensitivity and accuracy are improved.

**Table 1:** The detection methods of miRNAs based on portable instruments.

Methods	miRNA	Detection Limit	Samples	Time	Reference
Personal glucose meter	miR-21	0.41 nM/ 1 million cells	synthesized miR-21/A549 cell lysates	<2 h	[21]
	miR-21	10 fM	synthesized miR-21	<2 h	[23]
	miR-21 miRNA205	2.4 pM 1.1 pM	synthesized miR-21 synthesized miRNA205	<3 h	[26]
	miR-21	3.65 nM	synthesized miR-21 clinical serum samples from cancer patients	2 h	[22]
	miR-21	60 pM 3 × 10^6^ cells/mL	synthesized miR-21 MCF-7, A549 and HeLa cell lysates	<3 h	[24]
	miR-21	68.08 fM	synthesized miR-21 urine samples from DIKI mice	1.5 h	[25]
	miRNA-155	0.36 fM	synthesized miRNA-155	>5 h	[27]
	miR-21, miR-335, miR-155, and miR-122	0.325 fmol	synthesized miRNAs extract from HeLa, HepG2, MCF-7, and L02 Cells	6 h	[28]
Thermometer	miR-21	7.8 nM	synthesized miR-21 HeLa cell lysate	Not mentioned	[35]
	miRNA-141	0.5 pM	synthesized miRNA-141	>8 h	[36]
Pressure meter	miR-21	7.6 fM 100 cells	synthesized miR-21 A549, MCF-7, HepG2 and HL-7702 cells	20 min	[38]
	miR-21	10 pM	Serum	0.5 h	[39]
Portable fluorometer	miR-574-5p	2 ng/μL	RNA extract from 5XFAD mice	>3 h	[40]
Capillary force meter	miR-21	10 nM	Human serum	1 h	[43]
	miR-21		MCF-7 cell line	25 min	[44]
Smartphone	miR-133a	0.3 pM	synthesized miR-133a in serum	>5 h	[50]
	miRNA-499, miRNA-133a	10 fM	synthesized miR-133a in serum	13 h	[51]
	let-7a	1.7 fM	synthesized let-7a human serum	2.75 h	[56]
	miR-133a, miR-499	1 fM	Synthesized miRNAs human serum	-	[52]
	miR-21, let-7a	fM	Synthesized miRNAs human serum	<2 h	[49]
	miR-21	1.43 pM	Synthesized miR-21 human serum, urine	0.5 h	[58]
	miR-224	1.6 fM	Synthesized miR-224 human plasma	<4.5 h	[59]
	miR-21	100 fM 500 cells	Synthesized miR-21 MCF-7 and L02 cells	>1 h	[57]

### 2.2. Visual Detection of miRNAs Based on Colorimetry

Visual detection is particularly attractive for POCT because the readout can be read with the naked eye with no need for instruments. In this section, we summarize recent advances in the visual detection of miRNAs, mainly focusing on colorimetric methods. We provide an all-sided discussion of the principles of the methods and rationally evaluate the applicability of these visual detection methods for early diagnosis based on miRNA detection. A summary of the reported POCT for miRNAs based on colorimetric methods is presented below (Table 2).

Colorimetric assays provide qualitative or quantitative measurement of targets by measuring color changes with no need for special instruments. Detecting a change in color can be used to determine the presence or absence of a target or even to determine its amount range. As color changes can be conveniently judged by the naked eye, colorimetric assays have attracted increasing attention for POCT for miRNAs [12]. Herein, we summarize the colorimetric methods for the detection of miRNAs, mainly focusing on color change based on gold nanoparticle (AuNP) aggregation or disaggregation and enzymatic chromogenic reactions that catalyze the color change of substrates.

#### 2.2.1. Colorimetric Detection Based on Au-NPs

In 1996, Mirkin et al. first reported that the DNA-driven self-assembly of DNA-modified gold nanoparticles (DNA-GNPs) caused an obvious color change in solutions. In this study, the target DNA hybrid with two grafted DNA molecules was immobilized on DNA-GNPs, which drove the DNA-GNPs to assemble into aggregates. Since then, DNA-GNPs have attracted increasing attention in the development of colorimetric methods for POCT [60]. In principle, localized surface plasmon resonance (LSPR) leads to a blueshift in the aggregation of Au-NPs, and non-crosslinking and crosslinking hybridization are the two main strategies used in Au-NP aggregation [61].

**Figure 4:**
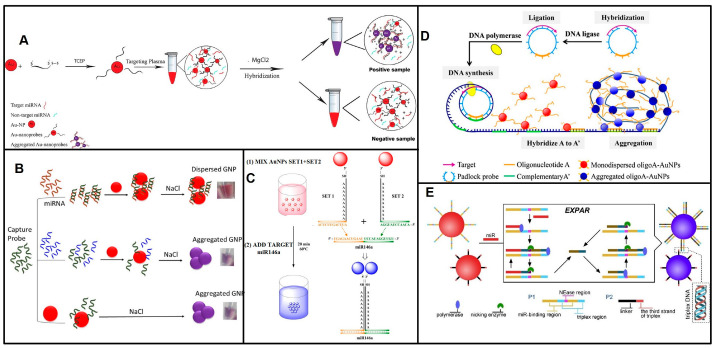
Principles of colorimetric detection of miRNAs based on Au-NPs. (**A**) Schematic of detection of miRNAs based on Au-NPs non-crosslinking aggregation due to the salting-out effect. Reproduced with permission from Reference [63]. Copyright (2021), with permission from Elsevier. (**B**) Schematic of detection of miRNAs based on target binding improve the stability of Au-NPs preventing the aggregation. Reproduced with permission from Reference [67]. Copyright (2021), with permission from Elsevier. (**C**) Schematic of detection of miRNAs based on RNA-functionalized Au-NPs crosslinking aggregation. Reproduced with permission from Reference [76]. Copyright (2020), with permission from Elsevier. (**D**) Schematic of miRNA-triggered RCA products lead to Au-NP aggregation. Reproduced with permission from Reference [81]. Copyright (2017), with permission from Elsevier. (**E**) Schematic of detection of miRNA based on EXPAR and triplex DNA lead to Au-NPs aggregation. Reproduced with permission from Reference [84]. Copyright (2020), with permission from Elsevier.

The non-crosslinking hybridization method is of greater interest than the crosslinking method due to its fast response, simplicity, and convenience. The first distinct mechanisms in non-crosslinking aggregation were reported by Sato and colleagues, who identified a short ssDNA using non-crosslinking aggregation in 2003 [62]. The hybridization of the target 15 nt ssDNA with 15 mer DNA immobilized on Au-NPs induced Au-NP aggregation due to the salting-out effect. The color change based on Au-NP aggregation can be detected in less than 3 min. Based on this mechanism, Asma Hamidi and coworkers detected miR-21 and miR-155 in plasma from cancerous patients (Figure 4A) [63]. They analyzed thirty samples, and a high concentration of miR-21 was detected in stomach, colon, breast, esophagus, sarcoma, diaphragm, prostate, bladder, and lung samples, while miR-155 demonstrated high expression in colon, breast, lung, diaphragm, and esophagus samples. As little as 5 ng μL^−1^ of miRNAs can be measured. Maria Pitou et al. also reported the easy and rapid detection of miR-93 in blood samples of osteoarthritic patients based on this non-crosslinking aggregation strategy [64]. Another method of non-crosslinking aggregation was first reported by Baptista et al. [65]. Unlike aggregation due to the salting-out effect, the target sequence binding to Au-NPs improved its stability and prevented a blueshift. miR-21 and miR-155 extracts from cancer cell lines, the osteosarcoma biomarker miRNA-195, the urinary microRNA-210-3p from bladder cancer patients, and urinary miRNAs (miR-210 and miR-34a) from diabetic nephropathy patients were measured using this non-crosslinking aggregation method (Figure 4B) [66,67,68,69]. The last mechanism of non-crosslinking aggregation was based on the ssDNA or the sticky end of harpin DNA closely absorbed on Au-NPs to prevent salt-induced Au-NP aggregation, while the negatively charged duplex DNA moved away from the Au-NPs, causing the naked Au-NPs to aggregate and appear blueshifted. Several groups reported label-free, enzyme-free, and immobilization-free miRNA strategies, in which the target miRNA induced ssDNA or harpin DNA to transform into duplexes, leading to Au-NP aggregation [70,71,72,73]. Compared to crosslinking aggregation, non-crosslinking aggregation has lower sensitivity.

The non-crosslinking hybridization method is of greater interest than the crosslinking method due to its fast response, simplicity, and convenience. The first distinct mechanisms in non-crosslinking aggregation were reported by Sato and colleagues, who identified a short ssDNA using non-crosslinking aggregation in 2003 [62]. The hybridization of the target 15 nt ssDNA with 15 mer DNA immobilized on Au-NPs induced Au-NP aggregation due to the salting-out effect. The color change based on Au-NP aggregation can be detected in less than 3 min. Based on this mechanism, Asma Hamidi and coworkers detected miR-21 and miR-155 in plasma from cancerous patients (Figure 4A) [63]. They analyzed thirty samples, and a high concentration of miR-21 was detected in stomach, colon, breast, esophagus, sarcoma, diaphragm, prostate, bladder, and lung samples, while miR-155 demonstrated high expression in colon, breast, lung, diaphragm, and esophagus samples. As little as 5 ng μL^−1^ of miRNAs can be measured. Maria Pitou et al. also reported the easy and rapid detection of miR-93 in blood samples of osteoarthritic patients based on this non-crosslinking aggregation strategy [64]. Another method of non-crosslinking aggregation was first reported by Baptista et al. [65]. Unlike aggregation due to the salting-out effect, the target sequence binding to Au-NPs improved its stability and prevented a blueshift. miR-21 and miR-155 extracts from cancer cell lines, the osteosarcoma biomarker miRNA-195, the urinary microRNA-210-3p from bladder cancer patients, and urinary miRNAs (miR-210 and miR-34a) from diabetic nephropathy patients were measured using this non-crosslinking aggregation method (Figure 4B) [66,67,68,69]. The last mechanism of non-crosslinking aggregation was based on the ssDNA or the sticky end of harpin DNA closely absorbed on Au-NPs to prevent salt-induced Au-NP aggregation, while the negatively charged duplex DNA moved away from the Au-NPs, causing the naked Au-NPs to aggregate and appear blueshifted. Several groups reported label-free, enzyme-free, and immobilization-free miRNA strategies, in which the target miRNA induced ssDNA or harpin DNA to transform into duplexes, leading to Au-NP aggregation [70,71,72,73]. Compared to crosslinking aggregation, non-crosslinking aggregation has lower sensitivity.

The crosslinking Au-NP aggregation method offers a reliable and effective solution for detecting nucleic acid sequences. By utilizing two DNA/RNA-modified Au-NPs with complementary sequences to two separate halves of the target nucleic acid, accurate detection is ensured without the need for complex equipment or procedures. The hybridization of these nanoprobes with the target DNA leads to Au-NP aggregation and a blueshift in the LSPR spectrum, providing a clear indication of the presence of the desired nucleic acid sequence [74]. Based on this principle, a miRNA molecule cross-links two Au-NPs together through a sandwich hybridization reaction. Jun Cai and coworkers detected miR-148a in gastric cancer samples, and a nanomolar level of miR-148a was detected with the colorimetric method [75]. miR-146a, a possible biomarker of mastitis, was also measured at the nM level using a similar strategy (Figure 4C) [76]. Motoi Oishi and Satomi Sugiyama developed miRNA-triggered GNP/MB-composite disaggregation allowing the detection of miRNAs with the naked eye, achieving a detection limit of 10 pM [77]. Jong-Souk Yeo et al. designed a nanoplasmonic core-satellite assembly strategy to detect 1 pM to 10 μM miR-21 [78]. To improve the sensitivity, the crosslinking aggregation strategy was coupled with the nucleic acid amplification method. Bang-Ce Ye et al. developed a DSN-assisted crosslinking aggregation strategy and detected pM-level miRNAs [79]. Yongmei Yin et al. developed a strategy of entropy-driven amplification (EDA) coupled with Nb.BbvCl-assisted Au-NP aggregation and successfully detected as little as 10 fM let-7a [80]. Yifu Guan and coworkers utilized target miRNA to initiate rolling circle amplification (RCA), and the hybridization of RCA products with the DNA immobilized on Au-NPs led to aggregation and blueshift, achieving a 0.13 pM detection limit (Figure 4D) [81]. Enzyme-free hybridization chain reaction (HCR) was also introduced into colorimetric detection-based on Au-NPs. The target miRNA released the initiator sequence, and the initiator triggered the HCR between the hairpin DNA modified on Au-NPs [82]. EXPAR, with the advantages of high amplification efficiency and speed, is attractive for crosslinking aggregation strategies, and the target miRNA-induced EXPAR products act as DNA linkers for Au-NP aggregation. This EXPAR-assisted crosslinking aggregation enabled the detection of aM-level miRNA targets (Figure 4E) [83,84] (Table 2). Bang-Ce Ye et al. reported that the target miRNA triggered the EXPAR and released the phosphorothioate-modified polyA from Au-NPs. Then, the naked Au-NPs aggregated in high-salt solution and appeared blueshifted, and the proposed method provided a detection limit of approximately 100 fM when read with the naked eye [85]. On the other hand, CRISPR-CasCas12a/Cas13a assisted the degradation of linker DNA in the presence of the target miRNA, resulting in Au-NP disaggregation, and colorimetric methods detected 500 fM miR-17 and 1 fM miR-143 from clinical samples of prostate cancer [86,87]. Recently, the Guo group developed a colorimetric method employing the crosslinking-aggregation of DNA Au-NPs and an entropy-driven dynamic DNA network (EDN), in which the target miRNAs triggered the release of converter DNA through the EDN, and converter DNA further triggered DNA Au-NP release from the hydrogel film. The color change of the DNA Au-NP hydrogel film and solution in the testing tube could determine the number of miRNAs [88]. Colorimetric assays using DNA-GNPs have attracted much attention as a visual detection platform for POCT; however, the complex and time-consuming preparation of DNA or RNA functional nanoparticles has hindered their practical usage in clinical diagnostics.

#### 2.2.2. Colorimetric Method Based on Enzymatic Chromogenic Reactions

**Figure 5:**
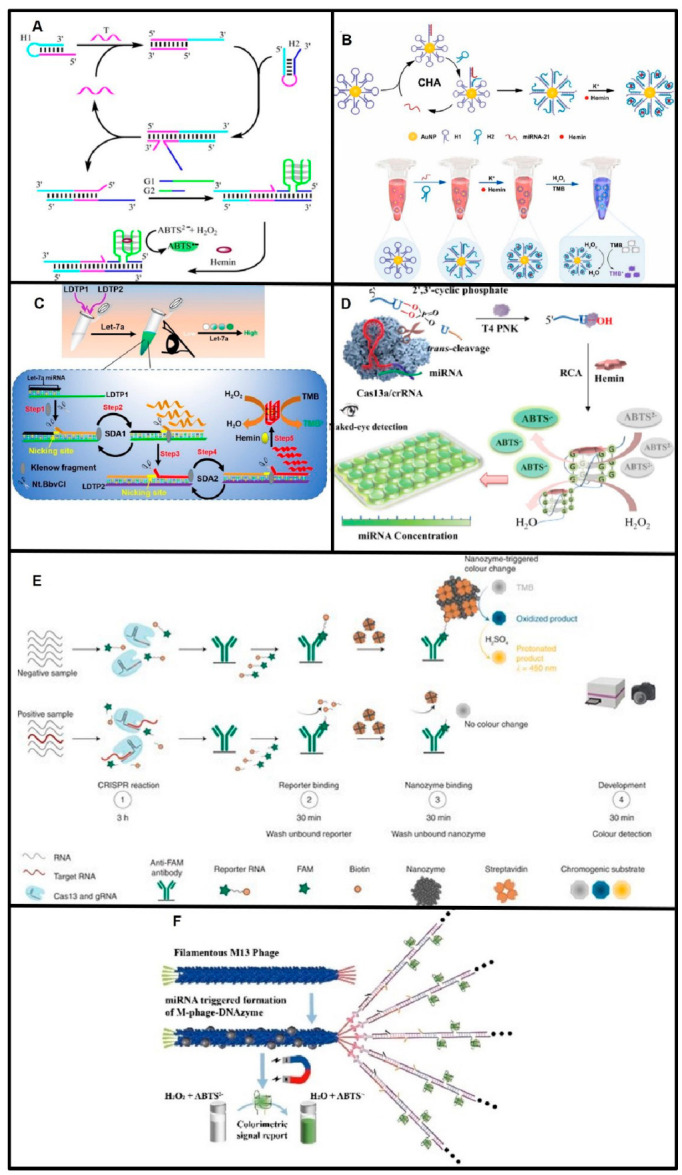
Principles of colorimetric detection of miRNAs based on enzymatic chromogenic reactions. (**A**) Schematic of CHA coupled with HCR to assemble G-quadruplex/Hemin DNAzyme. Reproduced with permission from Reference [92]. Copyright (2016), with permission from Elsevier. (**B**) Schematic of miRNA triggered self-assembled G-quadruplex/hemin DNAzyme. Reproduced with permission from Reference [105]. Copyright (2023), with permission from Elsevier. (**C**) Schematic of miRNA-triggered nicking enzyme-assisted self-assembled G-quadruplex/hemin DNAzyme. Reproduced with permission from Reference [108]. Copyright (2023), with permission from Elsevier. (**D**) Schematic of miRNA-triggered RCA to produced numerus G-quadruplex/Hemin DNAzymes. Reproduced with permission from Reference [97]. Copyright (2021), American Chemical Society. (**E**) Schematic of colorimetric detection of miRNAs based on nanozyme-catalyzed CRISPR assay. Reproduced with permission from Reference [118]. Copyright (2022), with permission from Elsevier. (**F**) Schematic of miRNA-triggered formation of G-quadruplex/Hemin DNAzyme fibers. Reproduced with permission from Reference [109]. Copyright (2022), John Wiley & Sons, Inc.

Enzymatic chromogenic reactions are widely used in colorimetric methods because they are stable and easy to operate. The G-quadruplex/hemin complex is currently a popular DNAzyme in colorimetric methods [89]. G-quadruplexes are four-stranded structures formed by tandem repeat G-rich sequences, and G-quadruplex/hemin is an HRP-mimic DNAzyme [90]. In the presence of H_2_O_2_, G-quadruplex/hemin DNAzyme can catalyze the oxidation of TMB or ABTS, resulting in a significant color change of substrates [90,91]. G-quadruplex/hemin DNAzyme holds great advantages in stability and easy preparation, making it a decisive tool for colorimetric readouts. G-quadruplex/hemin DNAzyme is widely used in miRNA colorimetric detection. Wu and coworkers designed CHA coupled with an HCR sensing strategy to produce numerous G-quadruplexes: the G-quadruplexes bind with hemin to catalyze ABTS, turning it green. ABTS^−^. This proposed method showed a detection limit of 7.4 fM (Figure 5A) [92]. Several other studies also utilized CHA coupled with the HCR sensing strategy [93,94]. Hosseinzadeh et al. reported a triple amplification strategy to detect miR-21, in which miR-21 triggered the formation of G-quadruplex DNAzymes to give a colorimetric signal [95]. Recent distinct strategies for G-quadruplex DNAzyme production include (1) target miRNA-triggered RCA to produce numerous G-quadruplex DNAzymes (Figure 5D) [96,97]; (2) miRNA-triggered RCA coupled with nicking enzyme assistance to produce G-quadruplex DNAzymes [98,99], (3) miRNA-induced toehold-mediated strand displacement reactions to release G-quadruplexes (Figure 5B) [100,101,102,103,104,105]; (4) nicking enzyme-assisted strand displacement amplification (Figure 5C) [106,107,108]; (5) target miRNA-triggered HCR to form long G-quadruplex DNAzyme chains [109]; and (6) DSN-assisted G-quadruplex DNAzyme release [110,111]. Beyond these, there are also some special colorimetric assay strategies based on G-quadruplexes. Melika Agahi and Mahdi Rahaie designed a split G-quadruplex-containing tweezer to detect dual miRNA markers [112]. Ling Lan and coworkers reported a G-quadruplex/Hemin DNAzyme blocking strategy to detect microRNA, in which G-quadruplex formation was hindered by the target miRNA through primer extension [113]. The Mao group reported that target miRNAs triggered the formation of G-quadruplex/Hemin DNAzyme fibers, which could serve as colorimetric signal reports (Figure 5F) [109]. Colorimetric assays can also be based on other chromogenic enzymes, such as alkaline phosphatase (AP) [114], DNA-templated copper nanoclusters (DNA-CuNCs) [115], glucose oxidase (GOx) [116], HRP [117], and nanozymes. Marta Broto et al. developed a Pt@Au nanozyme-based colorimetric assay for miRNA. The Pt@Au nanozymes were captured through an RNA linker and catalyzed the oxidation of TMB, resulting in a color change. In the presence of the target miRNA, the Cas13 enzyme cleaved the RNA linker, resulting in no color change (Figure 5E) [118].

Compared with colorimetric detection based on Au-NPs, the enzymatic chromogenic reactions strategies often take much more time, and both showed high sensitivity (Table 2). However, one major drawback of colorimetry is that it cannot accurately quantify miRNAs through color changes and requires other instruments, such as spectrometers and smartphones, to perform quantitative analysis.

**Table 2:** The detection methods of miRNAs based on visual methods.

Methods	miRNA	Detection Limit	Samples	Time	Reference
Colorimetric detection based on Au-NPs	miR-21 miR-155	5 ng μL^−1^	Plasma	<3 min	[63]
	miR-93 miR-223	-	Human serum	-	[64]
	miR-34a miR-210	50 ng μL^−1^	Urine	<20 min	[66]
	miR-195	40 fM	Human serum	10 min	[67]
	miR-210-3p	10 pM	Urine	20 min	[68]
	miR-21 miR-155	1 ng μL^−1^	Multiple cancerous cell lines and primary fibroblast	<10 min	[69]
	miR-21 miR-141	3 pM	Synthesized miRNA human serum samples	<5 h	[70]
	miR-137	0.5 nM	Plasma	1 min	[72]
	miR-146a	5 nM	Raw cow milk	20 min	[76]
	let-7a	0.13 pM	A549 cells	50 min	[81]
	miR-148a	1.9 nM	Plasma	5 min	[75]
	miR-122	16 pM	Cancerous cell lines	2 h	[79]
	let-7a	3.13 fM	Human serum	1 h	[80]
	miR-203	10 pM	MCF-7 cells	-	[82]
	miR-21	0.23 fM	HeLa, MCF-7, AGS cells	0.5 h	[84]
	let-7a	4.176 aM	Synthesized let-7a	1 h	[83]
	miR-221-3p	46 fM	BEL-7404, MDA-MB231, HeLa, and 22Rv1cells	1 h	[85]
	miR-143	1 fM	Synthesized miR-143 Prostate cancer cell lines VCaP, LNCaP, Du145, and PC-3	>1.5 h	[87]
Colorimetric detection based on enzymatic chromogenic reactions	let-7a	7.4 fM	Synthesized let-7a	2.5 h	[92]
	miR-122	0.15 aM	Serum	5 min	[93]
	miR-21	0.2 pM	Serum	50 min	[94]
	miR-21	1 aM	Serum	<4 h	[95]
	Let-7a	34 fM	A549 cells	4 h	[96]
	miR-10b	1 fM	Serum and cell extracts	20 min	[97]
	miR-141	0.48 nM	Serum	>3 h	[100]
	miR-21	1 pM	Serum	150 min	[102]
	miR-141	0.5 pM	Prostate cancer cells	210 min	[104]
	miR-21	90.3 fM	Serum	<1.5 h	[105]
	miR-21, miR-17	1.7 fM	MCF-7	80 min	[106]
	let-7a	0.1 nM	Serum	3 min	[108]
	miR-21	44.76 fM	Exosome	2 h	[111]
	miR-21, miR-155	0.38 nM	Blood	>1 h	[112]
	miR-21	4.5 nM	MCF-7 and serum	130 min	[113]
	miR-21	5 fM	Plasma sample Cancer cells Tumor tissues	>6.5 h	[109]
	miR-155	0.6 pM	Plasma	15 min	[115]
	miR-205, miR-944	36.4 fM	Serum	>2 h	[116]
	miR-155	31.8 fM	Serum	1 h	[117]
	miR-223 miR-143	20 pM	Synthesized miR-223 iPSCs and CMs	3.5 h	[118]

## 3. Conclusions and Future Perspectives

miRNAs are a type of small noncoding RNA that play crucial roles in the regulation of gene expression during development and disease progression by acting as post-transcriptional regulators. Studies have proven differential expression between diseased and healthy individuals, and miRNAs are stable in blood, saliva, and urine. These indicate that miRNAs can serve as promising noninvasive biomarkers for the diagnosis and prognosis of various diseases. Many studies have evaluated miRNA-based diagnostic tests for clinical use in many diseases, such as cancers. The usage of miRNAs as biomarkers has the potential to revolutionize the field of disease diagnosis, leading to earlier and more accurate diagnoses.

The conventional methods of detecting miRNAs, including northern blotting, real-time PCR, microarray and RNA-seq, have been widely used and validated. However, these methods also have limitations. For example, northern blotting and real-time PCR are labor-intensive and require specialized equipment. Microarray and RNA-seq are expensive and require complex data analysis, and these approaches for miRNA detection are performed in a professional laboratory, which is challenging for the application of miRNA detection in clinical practice. The huge demand for the improvement of health and safety has driven sensors to evolve rapidly [119,120,121,122]. Recent advances in POCT for miRNAs have shown promise in overcoming some of these challenges and have the potential to revolutionize miRNA detection in the future. Widely available pocket-size devices and visual detection play major roles in POCT. In this review, we mainly focused on summarizing the research progress in POCT for miRNAs based on portable instruments and visual readout methods and compared the design of POCT methods based on portable instruments and visual detection for miRNAs.

In the miRNA detection based on PGM, the release or trapping of invertase, amylase, or sucrase, which operates in the presence of miRNA, results in a glucose concentration proportional to the level of target miRNA. In addition, thermometers measure temperature change representing the presence and concentration of a target miRNA. A smartphone’s built-in camera or connected external sensors could collect chemiluminescence (CL)/bioluminescence (BL) or fluorescence signals proportional to the level of target miRNA; a smartphone can also share data with internet connections and achieve the automatic and rapid analysis of information.

The POCT for miRNA detection based on PGM, thermometers, and smartphones has the advantage of using portable instruments that are widely applied as home-use devices; it will be suitable for resource-limited areas, or even for self-testing, which assesses the risk of related diseases in a household’s devices without relying on sophisticated equipment or professional operation. Similarly, the visual detection of miRNAs based on colorimetry can also achieve signal reading without the need for complex operations with sophisticated instruments. Colorimetric methods sensed the miRNA level according to the color change based on gold nanoparticle (AuNP) aggregation or disaggregation and enzymatic chromogenic reactions that catalyze the color change of substrates.

There is no doubt that miRNA detection using portable instruments and visual detection has made significant progress over the years. Recent advances in portable instrumentation and visual detection have increased the sensitivity, specificity, and accuracy of miRNA detection, making it more promising for clinical applications. These methods offer rapid detection of miRNAs and are useful for low-resource settings where sophisticated instrumentation is not available. The use of POCT devices and visually read miRNA detection methods can help diagnose diseases, identify high-risk patients for further assessment, and facilitate the monitoring of disease progression and treatment efficacy. The progress made in miRNA detection using POCT devices and visual detection methods has paved the way for the development of new diagnostic approaches in clinical settings. As new technologies emerge, it is expected that miRNA detection and analysis will continue to evolve and become more accessible and affordable for routine clinical use. The emerging POCT assays for miRNAs based on capillary force meters, pressure meters, portable fluorometers, PGMs, and thermometers are still at the very early stage, and most of them reported detection of the synthesized or extracted miRNAs. While POCT assays for miRNAs based on smartphones showed higher sensitivity and stronger anti-interference ability than the others, as it was widely reported in the detection of clinical serum samples. Colorimetric assays provide measurement of the targets by measuring color changes with no need for special instruments. One major drawback of colorimetry is that it cannot accurately quantify miRNAs through color changes and requires other instruments, such as spectrometers and smartphones, to perform quantitative analysis.

However, miRNA-based POCT assays hold great potential for clinical applications, though several challenges need to be overcome to make them a reality. Firstly, the preparation of DNA/RNA immobilized Au or magnetic nanoparticles, as well as other nanomaterials used in these methods, is relatively complex and time-consuming, even if some materials are customized. Therefore, the development of easily preparable materials ensures the stability, repeatability, and efficiency of the detection methods. The simple and highly efficient “click” chemistry and the exploration on noncovalent interactions between the DNA/RNA and MBs or Au-nanoparticles could enable new possibilities in providing potential solutions to addressing these challenges [29,30]. Second, the standardization of miRNA quantification in POCT methods is crucial for the implementation of miRNA-based diagnostic tests. However, there is currently a lack of consensus on the optimal method for normalizing miRNA levels across different studies, which results in ambiguous data explanations, controversial conclusions, and erroneous predictions. POCT of miRNAs with spike-in would be helpful for normalizing miRNA levels like the processing of single-cell microRNA sequencing [123]. Lastly, and most importantly, most POCT methods have been validated with a single synthetic miRNA or purified from biological samples. However, in practice, biological samples, such as blood, saliva, and urine, are complex, and a single miRNA often fails to meet clinical diagnostic needs. Therefore, it is important to develop multichannel POCT biosensors for the detection of multiple miRNA biomarkers and set up a “sample-in-answer-out” microfluidic system for miRNA-based diagnostic tests. Multichannel POCT biosensors could meet the clinical diagnostic needs and perform the patient miRNA profile to determine the miRNA signatures. Multichannel POCT biosensors save time and cost and decrease the risks for error.

POCT of miRNAs is promising and widely used in the field of disease diagnosis, but it is necessary to simplify and pull together the process of miRNA purification, amplification, and signal transduction. This review introduced the development status of point-of-care testing of microRNAs based on portable instruments and visual detection and summarized the advantages and disadvantages of the common detection modes based on these methods, in order to provide a guide for the development of more accurate, specific, and sensitive miRNA POCT methods.

## Data Availability

Not applicable.
